# Efficacy of Morphine Combined with Mechanical Ventilation in the Treatment of Heart Failure with Cardiac Magnetic Resonance Imaging under Artificial Intelligence Algorithms

**DOI:** 10.1155/2022/1732915

**Published:** 2022-02-25

**Authors:** Zhihai Geng, Bolun Chen, Qiang Li, Xi Han, Xuelian Zhu

**Affiliations:** ^1^Department of Anesthesiology, First Affiliated Hospital of Jiamusi University, Jiamusi 154003, Heilongjiang, China; ^2^Department of Anesthesiology, Jiansanjiang Hospital of Beidahuang Group, Jiamusi 156399, Heilongjiang, China; ^3^Department of Anatomy, School of Basic Medical Sciences, Jiamusi University, Jiamusi 154007, Heilongjiang, China

## Abstract

This study was aimed at exploring the efficacy of morphine combined with mechanical ventilation in the treatment of heart failure with artificial intelligence algorithms. The cardiac magnetic resonance imaging (MRI) under the watershed segmentation algorithm was proposed, and the local grayscale clustering watershed (LGCW) model was designed in this study. A total of 136 patients with acute left heart failure were taken as the research objects and randomly divided into the control group (conventional treatment) and the experimental group (morphine combined with mechanical ventilation), with 68 cases in each group. The left ventricular end-diastolic diameter (LVEDD), left ventricular end-systolic diameter (LVESD), left ventricular ejection fraction (LVEF), N-terminal pro-brain natriuretic peptide (NT-proBNP), arterial partial pressure of oxygen (PaO_2_), and arterial partial pressure of carbon dioxide (PaCO_2_) were observed. The results showed that the mean absolute deviation (MAD) and maximum mean absolute deviation (max-MAD) of the LGCW model were lower than those of the fuzzy k-nearest neighbor (FKNN) algorithm and local gray-scale clustering model (LGSCm). The Dice metric was also significantly higher than that of other algorithms with statistically significant differences (*P* < 0.05). After treatment, LVEDD, LVESD, and NT-proBNP of patients in the experimental group were significantly lower than those in the control group, and LVEF in the experimental group was higher than that in the control group (*P* < 0.05). PaO_2_ of patients in the experimental group was also significantly higher than that in the control group (*P* < 0.05). It suggested that the LGCW model had a better segmentation effect, and morphine combined with mechanical ventilation gave a better clinical efficacy in the treatment of acute left heart failure, improving the patients' cardiac function and arterial blood gas effectively.

## 1. Introduction

Heart failure is the ventricular insufficiency caused by structural or functional diseases of the heart. It is usually classified into acute heart failure and chronic heart failure, and the former can be a sign of aggravation of the latter [1–3]. Heart failure is more common among the elderly aged more than 65 years, most of which are chronic heart failure [4]. Meanwhile, heart failure can also be divided into left heart failure and right heart failure. Acute left heart failure is clinically the most common, which manifests as severe dyspnea, orthopnea, expectoration with pink foamy sputum, dysphoria, fear, and so on. Hypoxemia may occur in patients with heart failure; for some severe cases, it would occur complicated with acute pulmonary edema or cardiogenic shock. [5, 6]. Therefore, the key to the treatment of heart failure is to improve cardiopulmonary function and oxygenation of patients timely.

Mechanical ventilation is the main method for the treatment of acute left heart failure, which can be divided into noninvasive and invasive mechanical ventilations [7]. Invasive mechanical ventilation brings some complications and is not easy to be accepted by patients, while noninvasive positive pressure mechanical ventilation can achieve the effect of increased ventilation in the alveoli without trachea cannula [8, 9]. Some studies have found that noninvasive positive pressure mechanical ventilation is effective to reduce the work of breathing in patients with left heart failure, thereby reducing oxygen loss and improving the ratio of ventilation to blood flow [10]. However, patients with left heart failure are often complicated by unhealthy emotions such as irritability and fear, and poor tolerance to noninvasive ventilation, which greatly reduces the efficiency of mechanical ventilation and gives a poor efficacy [11]. Morphine is a commonly used drug for the treatment of acute left heart failure. It diminishes the angiectasis of the peripheral blood vessels by inhibiting the sympathetic nerves, so as to reduce the returned blood volume as well as the pressure load on the heart of patients [12]. Meanwhile, morphine has a good sedative effect to ease the irritability, fear, and more of patients, induce them to fall asleep, and reduce their oxygen consumption [13]. As a result, morphine can make patients with acute left heart failure adapt to mechanical ventilation better, reduce human-machine asynchrony, and improve the effect of mechanical ventilation and the clinical prognosis [14].

Cardiac magnetic resonance imaging (MRI) is accurate to measure ventricular volume and functions, and is considered to be the gold standard for noninvasive assessment of cardiac function. MRI can not only present the cardiac anatomical structure clearly, but also show the structure of the myocardium, the ventricular wall thickness, and the surrounding blood vessels. It plays an important role in the diagnosis, treatment, and prognostic evaluation of heart failure [15, 16]. However, in the process of cardiac MRI, the artifacts due to blood flow would lead to gray inhomogeneity, and factors like breathing and sternocleidomastoid muscle would also cause the target boundary to be unclear or even broken [17]. Artificial intelligence algorithms have shown superior performances in image segmentation and gradually have a cross-combination with medical imaging. Li et al. [18] used the golden-angle radial data for real-time exercise stress cardiac MRI reconstruction, detecting the increase in stroke volume and ejection fraction caused by exercise in a healthy heart. Ammar et al. [19] applied the full convolutional neural network to achieve cardiac MRI sequence segmentation as it can handle the pixel-level classification. They calculated three key structural volume characteristics effectively of the left ventricular cavity, right ventricular cavity, and left ventricular myocardium for disease prediction. A watershed-based cardiac MRI segmentation algorithm was proposed in this study for cardiac MRI imaging, and it was used to evaluate the efficacy of morphine combined with noninvasive mechanical ventilation in the treatment of acute left heart failure. To deal with the issues of cardiac MRI, a watershed-based cardiac MRI segmentation algorithm was proposed to evaluate the efficacy of morphine combined with noninvasive mechanical ventilation in the treatment of acute left heart failure.

In summary, improving cardiopulmonary function timely and preventing vital organ failure are the keys to the treatment of acute left heart failure. Noninvasive positive pressure ventilation helps to improve the ratio of ventilation to blood flow effectively, and morphine can make patients with acute left heart failure adapt to noninvasive mechanical ventilation better. Therefore, the watershed-based cardiac MRI segmentation algorithm was proposed in this study, together with the local gray-scale clustering model (LGSCm) introduced, for the efficacy evaluation of morphine combined with mechanical ventilation in the treatment of acute left heart failure. It was expected to provide a theoretical support for the clinical treatment of patients with acute left heart failure.

## 2. Materials and Methods

### 2.1. Research Objects and the Grouping

One hundred and thirty-six patients with acute left heart failure, who were admitted to the hospital from March 2018 to February 2020, were selected as the research objects. According to the random number table method, they were divided into the experimental group and the control group, with 68 people in each group. The general clinical information of the patients, including their name, gender, and age, was collected. This study was approved and supported by the ethics committee of hospital. All participant patients signed the written informed consents and volunteered to participate in this study.

Patients included met the following inclusion criteria. They were 18–73 years old, could receive mechanical ventilation treatment, and were in line with the clinical diagnosis of acute left heart failure. The exclusion criteria were described as follows. The patients had a weak spontaneous breathing, were complicated with vital organ function failure, or had a poor compliance. They had the upper respiratory tract obstruction or were severely allergic to the drugs used in the study.

### 2.2. Therapeutic Methods

The patients in the control group received conventional treatments, which mainly included sedation, diuresis, heart strengthening, blood vessel expansion, airway spasm relief, and oxygen inhalation. The oxygen flow rate was 5–10 L/min and was adjusted according to the specific conditions of patients. In addition to the above conventional treatment, ventilator was given to the patients in the experimental group to assist breathing. 5–10 mg morphine was also given through subcutaneous injection or intravenous infusion. After 1–2 hours, the dosage was adjusted according to the patients' specific conditions.

### 2.3. Cardiac MRI Examination

Cardiac MRI examination was performed. The scanning acquisition was completed at the end expiration when the patients held their breath. The true steady-state precession sequence with retrospective electrocardiograph-gating was applied to scan layer by layer from the cardiac base to the apex, and multiple layers of images were continuously collected, and 25 frames of images were collected in each cardiac cycle. The scanning parameters were set as follows. The layer thickness was 6-8 mm, the number of layers was 10–12, the time of repetition (TR) was 3.5 ms, the time of echo (TE) was 1.8 ms, the flip angle (FA) was 45°, and the field of view was 320 × 320. Two experienced radiologists in the field of cardiac radiology analyzed the collected images independently. If there was a difference in the comments of them, the final results were obtained after discussion between them.

### 2.4. Establishment of Local Grayscale Clustering Watershed (LGCW) Segmentation Model

**Figure 1:**
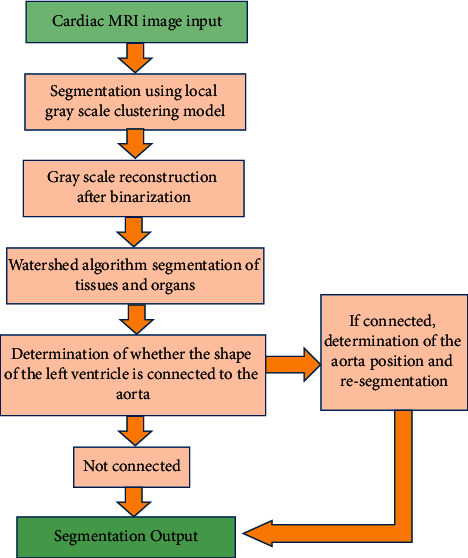
The segmentation flowchart of the LGCW model.

Watershed algorithm is commonly used in image segmentation, to deal with the issues such as blurred or missing image boundaries. It repairs the missing boundaries based on the existing boundaries of tissues and organs, to complete a closed contour boundary line so that tissues and organs are separated on the image. For a more accurate segmentation of noise-free cardiac MRI images, the LGSCm was introduced in this study [20]. Combined with the watershed algorithm, an LGCW segmentation model was designed. The segmentation process of the LGCW model is shown in Figure 1.

With the local gray-scale clustering criteria, a local area model in the local area of the image was obtained through LGSCm; thereby, the gray inhomogeneity was handled. *K(nm)* was assumed to represent the Gaussian kernel function, *P(m)* represented the image, *e(n)* represented the gray inhomogeneity feature of the approximated image, and *e(n)t*_*2*_ represented the clustering center of average local grayscale values. As *H* represented the Heaviside function, its specific energy function is defined as follows:

(1) $$\begin{array}{c} E=\int^{}\left(\int^{}K\left(n-m\right){\left(P\left(m\right)-e\left(n\right)t_{1}\right)}^{2}H\left(\varphi \left(m\right)\right)l_{m}\right)l_{n}+\int \left(\int K\left(n-m\right){\left(P\left(m\right)-e\left(n\right)t_{2}\right)}^{2}\left(1-H\left(\varphi \left(m\right)\right)l_{m}\right.\right)l_{n}. \end{array}$$

The LGSCm overcame the image noise and gray inhomogeneity, and segmented the tissues and organs of cardiac MRI images. However, it had the disadvantage of blurred or missing tissue boundaries. The watershed algorithm could solve this issue, so the LGCW segmentation model was designed by combining the two algorithms. The LGSCm was used for the cardiac MRI image segmentation, to obtain the initial contour at first. Then, the optimal solution was worked out via (1) iteratively, through which the contour line and the offset field were continuously corrected. After the gray inhomogeneity caused by the offset field and the noise were removed, the tissues and organs in the cardiac MRI image were segmented. The segmented cardiac MRI image was then binarized, and the threshold value within the contour area was fixed as 1, whereas the threshold value outside the area was 0. The Euclidean distance transform [21] was used to reconstruct the grayscale image, and the minimum grayscale areas were formed in different tissues and organs in the image. After that, the grayscale image was segmented through the watershed algorithm, to build a watershed between the tissues and organs so that the tissues and organs adhered to each other were divided. Finally, the accurate cardiac MRI segmentation was worked out. The segmentation was carried out in the experimental environment of MATLAB 2015a, CPU of 2.60 GHz, RAM of 3.0 GB, and Windows7 Professional.

### 2.5. Observation Indicators

**Figure 2:**
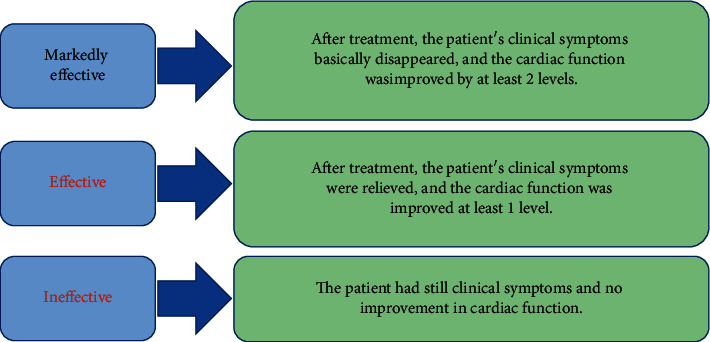
Evaluation criteria of the efficacies.

The clinical efficacy and cardiac functional indicators of patients in the two groups were compared, and the indictors included left ventricular end-diastolic diameter (LVEDD), left ventricular end-systolic diameter (LVESD), left ventricular ejection fraction (LVEF), and N-terminal pro-brain natriuretic peptide (NT-proBNP). Vital sign indicators of respiratory rate, systolic blood pressure, and diastolic blood pressure, and arterial blood gas indicators of arterial partial pressure of oxygen (PaO_2_), and arterial partial pressure of carbon dioxide (PaCO_2_) were also observed. The criteria of different efficacies are described in Figure 2.

The total effective rate is expressed as follows:

(2) $$\begin{array}{c} \text{total effective rate }\left(\text{\%}\right)=\frac{\text{significantly effective}\left(\text{n}\right)+\text{effective}\left(\text{n}\right)}{\text{total people}\left(\text{n}\right)}\times 100\text{\%.} \end{array}$$

The mean absolute deviation (MAD), maximum mean absolute deviation (max-MAD), and Dice metric (DM) were used to evaluate the performance differences among different algorithms. The MAD and max-MAD are expressed in the following equations:

(3) $$\begin{array}{c} \text{MAD}\left(K,L\right)=\frac{1}{2}\left(\frac{1}{x}\right.\left(\sum_{s=1}^{x}f\left(k_{x},L\right)+\frac{1}{r}\sum_{t=1}^{x}f\left(l_{t},K\right).\right. \end{array}$$

(4) $$\begin{array}{c} , \end{array}$$

where *f*(*k*_*x*_, *L*)=min‖*k*_*x*_ − *l*_*t*_‖, *f*(*l*_*t*_, *K*)=min‖*l*_*t*_ − *k*_*x*_‖, and *K*={*k*_1_, *k*_2_, *k*_3_ … *k*_*x*_} were the points on the contour of the automatically segmented image; whereas *L*={*l*_1_, *l*_2_, *l* … *l*_*x*_} was the point on the contour of the manual segmentation image. The smaller the MAD value, the smaller the average difference between the automatic segmentation and the manual segmentation. The smaller the max-MAD value, the smaller the maximum difference between the automatic and the manual segmentations. DM was to calculate the similarity between the automatic and the manual segmentations, and it was expressed in the following equation:

(5) $$\begin{array}{c} DM=\frac{2\left|Z\cap D\right|}{\left|Z\right|+\left|D\right|}, \end{array}$$

where *Z* is the area manually segmented and *D* is the area automatically segmented by the algorithm. The value of DM ranged from 0 to 1; the larger the value, the better the segmentation effect by the algorithm; the smaller the value, the poorer the algorithmic segmentation effect or of the segmentation error.

### 2.6. Statistical Analysis

The data were processed via SPSS 22.0. Normally distributed data were recorded in the form of mean ± standard deviation, and nonnormally distributed data were recorded in median (P25-P75). Normal distribution values between groups were compared by *t*-test, nonnormally distributed values were compared by rank sum test, and categorical variables were compared by *χ*^2^ test; *P* < 0.05 showed the difference was statistically significant.

## 3. Results

### 3.1. Performance Analysis of Algorithms

**Figure 3:**
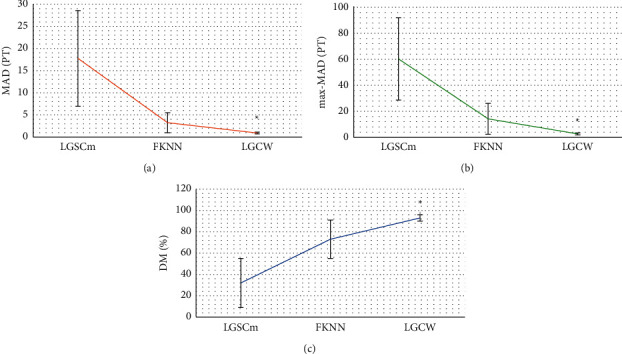
Performance analysis of the three algorithms. (a) The results of MAD; (b) results of max-MAD; (c) results of DM. ^*∗*^ indicates that the differences were statistically significant compared to those of the LGCW algorithm (*P* < 0.05).

As the fuzzy k-nearest neighbor (FKNN) algorithm [22] was introduced in this study, the MAD, max-MAD, and DM of the FKNN, LGSCm, and LGCW were compared and analyzed, with the results shown in Figure 3. The MAD values of LGSCm, FKNN, and LGCW were 17.76 ± 10.8PT, 3.24 ± 2.27PT, and 0.92 ± 0.19PT, respectively; the max-MAD values of them were 60.36 ± 31.64PT, 14.25 ± 11.87PT, and 2.65 ± 0.73PT, respectively; the DM values were 32 ± 23%, 73 ± 18%, and 93 ± 3%, respectively. As the values were compared with those of the LGCW model, there were statistically significant differences (*P* < 0.05). It was suggested that the segmentation of the LGCW model differed very little from the manual segmentation, with a strong robustness and high similarity.

### 3.2. Images of Segmentation

**Figure 4:**
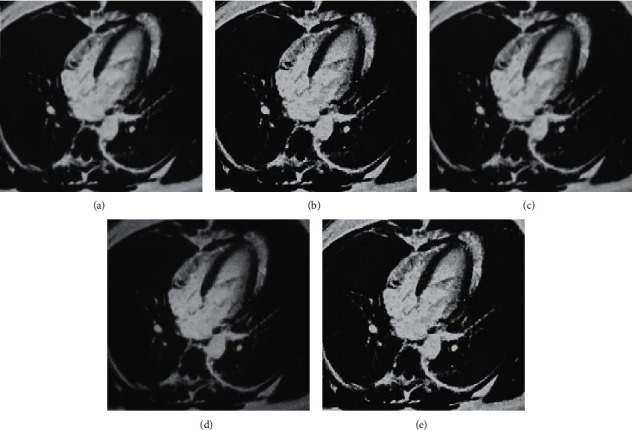
Segmentation results of three algorithms. (a) The image needed to be segmented; and (b–e) shows the images segmented by manual, LGSCm, FKNN, and LGCW algorithm, respectively.

The segmentation results of the three algorithms were compared, as shown in Figure 4. These images were the cardiac MRI images of a 67-year-old male patient. Compared with the manual segmentation, the LGCW model achieved the best segmentation effect, with the clearest boundary; the FKNN was the second.

### 3.3. General Clinical Data of the Patients

**Figure 5:**
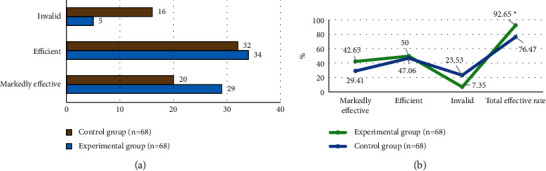
Comparison of clinical efficacy between the two groups. (a) The statistics of patient numbers with different efficacies. (b) The comparison of different efficacies. ^*∗*^ indicates that there was a statistically significant difference compared to the data of the experimental group (*P* < 0.05).

A total of 136 patients were included in the study. Their general clinical data are shown in Table 1. In the control group, there were 37 males and 31 females, aged 27–73 years, with an average age of 46.72 ± 12.4 years, the heart rate of 125.33 ± 6.15f/min^−1^, and the urine volume of 39±9 mL/h. In the experimental group of 36 males and 32 females, the patients were 26–72 years old with an average age of 47.21 ± 11.32 years, the heart rate of 126.24 ± 6.21 f/min^−1^, and the urine volume of 40 ± 10 mL/h; thus, there was no statistically significant difference (*P* > 0.05). The comparison is shown in Figure 5.

### 3.4. Comparison of Clinical Efficacy between Two Groups

The clinical efficacy on patients in the two groups was counted, as shown in Figure 5. In the control group, the patient number of the markedly effective, effective, and ineffective were counted as 20 (29.41%), 32 (47.06%), and 16 (23.53%), respectively; and the total effective rate was 76.47%. In the experimental group, there were 29 (42.65%), 34 (50%), and 5 (7.35%) patients were counted with the efficacy of the markedly effective, effective, and ineffective, respectively; and the total effective rate was 92.65%. The total effective rate of the experimental group was significantly higher than that of the control group, with a statistically significant difference (*P* < 0.05).

### 3.5. Comparison of Patients' Cardiac Function before and after Treatment

**Figure 6:**
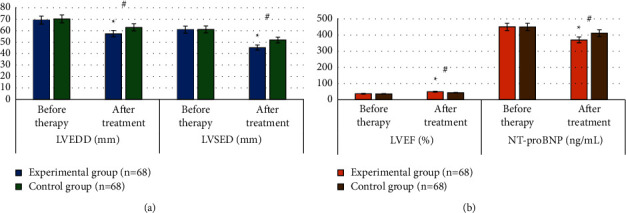
Comparison of cardiac function of patients before and after treatment. (a) The comparison of LVEDD and LVESD. (b) The comparison of LVEF and NT-proBNP. # indicates that the differences were statistically significant compared to the data before treatment in the same group, and ^*∗*^ indicates the statistically significant differences compared to the data of the control group (*P* < 0.05).

After treatment, the LVEDD, LVESD, and NT-proBNP of patients in both groups were significantly lower than those before treatment, and LVEF was significantly higher than those before treatment. LVEDD (57.36 ± 3.74 mm), LVESD (45.21 ± 6.24 mm), and NT-proBNP (369.85 ± 27.64 ng/mL) of patients in the experimental group after treatment were significantly lower than those in the control group. LVEF (50.05 ± 6.18%) in the experimental group after treatment was higher than that in the control group, showing statistically significant differences (*P* < 0.05). More details are shown in Figure 6.

### 3.6. Comparison of Vital Signs after Treatment

The vital signs of patients in the two groups after treatment are shown in Table 2. The respiratory rate of patients in the experimental group was 18.25 ± 1.27 f/min^−1^, the systolic blood pressure was 102.78 ± 4.81 mmHg, the diastolic blood pressure was 73.55 ± 4.55 mmHg, and the heart rate was 80.72 ± 4.26 f/min^−1^, which were all significantly smaller than those in the control group, with the statistically significant differences (*P* < 0.05).

### 3.7. Comparison of Arterial Blood Gas of Patients after Treatment

**Figure 7:**
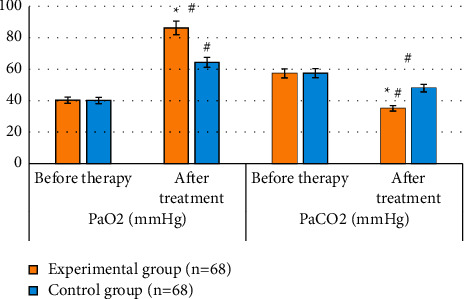
Comparison of arterial blood gas after treatment. Notes: # indicates that the differences in the same group were statistically significant compared to the data before treatment, and ^*∗*^ indicates that the differences compared to those in the control group were statistically significant (*P* < 0.05).

The arterial blood gas of patients in the two groups after treatment was compared and analyzed, as shown in Figure 7. The blood gas indicators of patients in both two groups were significantly different from those before treatment; the patients' PaO_2_ was 86.27 ± 20.35 mmHg in the experimental group after treatment, which was significantly higher than that of the control group; the PaCO_2_ of patients in the experimental group after treatment was 35.13 ± 6.81 mmHg, which was significantly lower than that of the control group, and the differences were all statistically significant (*P* < 0.05).

## 4. Discussion

Heart failure is a kind of ventricular insufficiency due to various predisposing factors in the heart. Its clinical symptoms include dyspnea, coughing, and lack of strength, and in severe cases, it may also cause pulmonary edema or cardiogenic shock [23]. Acute left heart failure is common among heart failures, and it is manifested as a sudden increase in pressure load on the left heart and in pulmonary circulation resistance. Clinically, mechanical ventilation is commonly applied in the treatment of acute left heart failure [24]. Masip [25] evaluated the role of noninvasive ventilation in acute heart failure. It is found that noninvasive ventilation can improve respiratory distress quickly, reduce the need for intubation in patients with acute cardiogenic pulmonary edema, and decrease the mortality rate. Therefore, the intelligent algorithm-based cardiac MRI was proposed to evaluate the efficacy of morphine combined with mechanical ventilation in the treatment of acute left heart failure in this study. The LGSCm and the watershed algorithm were introduced, and on these bases, the LGCW model was designed and compared with the FKNN as well as LGSCm algorithms. It was shown that the MAD and max-MAD of the LGCW segmentation model were significantly smaller than those of other algorithms, the DM value was much higher than that of other algorithms, and the differences were statistically significant (*P* < 0.05). This indicated that the segmentation by LGCW had a small difference from the manual segmentation, with a strong robustness and high similarity. Roy et al.[26] applied the watershed algorithm in segmentation of lung lesion images and proposed a multiview deep learning-driven iterative watershed algorithm, and finally, the similar results were obtained with those of this study.

Kawaguchi et al. [27] used morphine in the treatment of patients with advanced heart failure and drew a conclusion that applying morphine is feasible in the palliative care of patients with advanced heart failure complicated with refractory dyspnea. In this study, patients in the control group received conventional treatment, and patients in the experimental group were treated with morphine, which was to evaluate the efficacy of the two methods in the treatment of patients with acute left heart failure. The study found that the markedly effective rate, effective rate, ineffective rate, and total effective rate in the experimental group were 42.65%, 50%, 7.35%, and 92.65%, respectively. The total effective rate was significantly higher than that in the control group, and the difference was statistically significant (*P* < 0.05). It was indicated that morphine could alleviate the clinical symptoms effectively and improve cardiac function of patients, which was similar to the results of Johnson et al. [28]. After treatment, the LVEDD, LVESD, and NT-proBNP of patients in the two groups were significantly lower than those before treatment, and the LVEF was significantly higher than that before treatment; at the same time, the LVEDD, LVESD, and NT-proBNP of patients in the experimental group were significantly lower than those of the control group, and the LVEF of the experimental group was also significantly higher than that of the control group; the differences were statistically significant (*P* < 0.05). The vital signs of patients in the experimental group were close to normal values after treatment and were significantly lower than those in the control group with the statistically significant differences (*P* < 0.05). In the comparison of arterial blood gas, the PaO_2_ of patients in the experimental group was 86.27 ± 20.35 mmHg, which was obviously higher than that of the control group; PaCO_2_ was 35.13 ± 6.81 mmHg, which was significantly lower than that of the control group, showing statistically significant differences (*P* < 0.05). It was concluded that morphine combined with mechanical ventilation had a great the clinical efficacy in the treatment of acute left heart failure. The main reason was the sedative effect of morphine could maintain the patients' vital signs, reduce the stress responses, and make the patients more tolerant to mechanical ventilation, and then, the cardiac function and arterial blood gas were improved.

## 5. Conclusion

In this study, the efficacy of morphine combined with mechanical ventilation was researched in the treatment of acute left heart failure with the cardiac MRI based on the LGCW segmentation model. The FKNN and the LGSCm algorithms were also compared and analyzed; the clinical efficacy, the cardiac function, vital signs, and arterial blood gas were evaluated, as conventional treatments were applied in the control group and morphine was used in the experimental group. It was found from the results that the difference in segmentation effect between the LGCW model and manual segmentation was minimal, and the LGCW model had a strong robustness and high similarity. Morphine combined with mechanical ventilation in the treatment of acute left heart failure had a good clinical efficacy, and it could improve the cardiac function and arterial blood gas of patients effectively. However, the selected cases in this study were less relatively, which led to a lack of sample capacity, and follow-up investigations were not included, requiring further in-depth research. All in all, this study gave a theoretical support for the clinical treatment of patients with acute left heart failure.

## Figures and Tables

**Table 1:** General clinical data of patients in the two groups.

Groups	Gender		Age (years)	Heart rate (f/min^−1^)	Urine volume (mL/h)
	Male (*n*)	Female (*n*)			
Control group (*n* = 68)	31	37	46.72 ± 12.4	125.33 ± 6.15	39 ± 9
Experimental group (*n* = 68)	32	36	47.21 ± 11.32	126.24 ± 6.21	40 ± 10

**Table 2:** Comparison of vital signs of patients.

Groups	Respiratory rate (f/min^−1^)	Systolic blood pressure (mmHg)	Diastolic blood pressure (mmHg)	Heart rate (f/min^−1^)
Control group (*n* = 68)	20.23 ± 1.54^*∗*^	110.03 ± 6.33^*∗*^	80.62 ± 6.1^*∗*^	98.23 ± 3.79^*∗*^
Experimental group (*n* = 68)	18.25 ± 1.27	102.78 ± 4.81	73.55 ± 4.55	80.72 ± 4.26

*Note.* ^*∗*^ indicates that the differences were statistically significant compared to corresponding data in the experimental group (*P* < 0.05).

## Data Availability

The data used to support the findings of this study are available from the corresponding author upon request.
